# Expert-Rated Documentary Quality of AI-Assisted Hospital Discharge Reports: A Retrospective Paired Comparison with Physician-Written Reports

**DOI:** 10.3390/healthcare14152290

**Published:** 2026-07-29

**Authors:** Daniela Velásquez-Villegas, Toni Alonso Solís, Alex Trejo-Omeñaca, Xavier Serrano-Vinaixa, Michelle Cavariani Catta-Preta, Josep Monguet-Fierro, Ramon Romeu-Garcia, Beatriu Bayes-Genis, Carles Rubies-Feijoo, Esteve Llargués-Rocabruna

**Affiliations:** 1 Hospital General de Granollers, 08402 Granollers, Spain; dvelasquez@fphag.org (D.V.-V.); talonso@fphag.org (T.A.S.); rromeu@fphag.org (R.R.-G.);; 2 INNEX, 08800 Vilanova i La Geltrú, Spain; alex@innex.io (A.T.-O.); michelle@innex.io (M.C.C.-P.); josep@innex.io (J.M.-F.); 3 Department of Graphic Engineering and Design, Escola Politècnica Superior d’Enginyeria de Vilanova i la Geltrú (EPSEVG), Universitat Politècnica de Catalunya, 08800 Vilanova i La Geltrú, Spain; 4 Department of Medicine, Universitat Internacional de Catalunya, 08017 Barcelona, Spain

**Keywords:** artificial intelligence, large language models, hospital discharge report, clinical documentation, quality of care, data protection, GDPR, automated clinical notes

## Abstract

Background/Objectives: The hospital discharge report is a critical document for care continuity that generates a substantial administrative burden for clinicians. Generative artificial intelligence (AI) offers the potential to reduce this burden while improving documentary quality. This study aims to compare, under real-world conditions with a GDPR-oriented architecture based on prior local anonymisation, the quality of AI-assisted discharge reports (IAIA) against those drafted by the responsible physician (INF). Methods: A retrospective, paired, expert-evaluation study was conducted at a Spanish university hospital. One hundred and twenty consecutive clinical cases from nine departments were included (240 reports total). Each case was independently evaluated by one of ten primary care physicians using a structured rubric covering 13 clinical dimensions (ordinal scale 1–3) and a global rating scale (1–10). The Wilcoxon signed-rank test was applied to all paired comparisons; effect size was estimated using the paired rank-biserial correlation (r). Results: IAIA achieved a significantly higher overall mean rating than INF (8.14 vs. 7.30 out of 10; *p* < 0.0001; r ≈ 0.76, large effect). IAIA was nominally superior in 9 of 13 clinical dimensions; after Bonferroni correction for the 13 per-dimension comparisons, six of these differences remained statistically significant, with the largest gains in family history, principal diagnosis hierarchy, and structured listing of secondary diagnoses. INF retained an advantage only in allergies and intolerances (2.69 vs. 2.46; *p* = 0.002), where IAIA tended to use generic formulas. Three dimensions showed no significant difference (prior treatment, physical examination, procedures). Conclusions: AI-assisted discharge reports received higher expert-rated documentary quality scores in a non-blinded paired evaluation across most evaluated dimensions. The physician-written report retained an advantage only in the safety-critical allergy domain, where allergy information must not be inferred by the model but sourced from verified structured fields or explicitly flagged as pending physician validation, supporting the need for a supervised hybrid model in which AI generates the initial draft while the clinician mandatorily validates sensitive content. Prior local anonymisation constitutes a GDPR-oriented approach to generative AI deployment in European hospital settings, substantially reducing the risk of disclosure of identifiable clinical information.

## 1. Introduction

The hospital discharge report is the primary vehicle for clinical communication between inpatient and outpatient care, and its quality has direct consequences for medication safety, follow-up adherence, and 30-day readmission risk. More than fifteen years of research have consistently demonstrated that conventional discharge reports are often incomplete, arrive with delay, or fail to convey an operational post-discharge plan, with measurable adverse effects on care continuity and patient safety [1,2]. Their drafting also represents a significant administrative burden for clinicians, who must retrieve, structure, and re-synthesise information already available in the electronic health record (EHR).

Reducing this burden is one of the explicit objectives of the current generation of generative AI tools applied to clinical documentation. Large language models (LLMs) have demonstrated the ability to encode clinical knowledge and, when adapted to specific tasks, can achieve performance comparable or superior to that of medical experts in certain clinical text summarisation exercises [3,4,5]. This finding, however, was task-specific to the summarisation benchmarks reported by Van Veen and colleagues and does not necessarily generalise to hospital discharge report generation. Studies focusing specifically on AI-generated discharge summaries report a more mixed picture: feasibility comparisons have found AI-generated summaries reaching a quality level comparable to a junior physician’s [6], while blinded comparative studies have found AI-generated summaries similarly or equally preferred overall but less comprehensive and more error-prone than physician-written ones [7], and EHR-integrated deployments have found lower completeness in AI-generated summaries even though total quality scores did not differ significantly from physician-written ones [8]. These mixed and partly null findings temper the extent to which superiority demonstrated in other clinical text summarisation tasks can be assumed for discharge report generation specifically.

Early deployments of ambient AI scribes, AI-generated replies to patient inbox messages, and AI-generated patient visit summaries have shown improvements in clinical workflow [9,10,11]; however, publications in JAMA and the New England Journal of Medicine warn that fluent but ungrounded generation, hallucinated recommendations, and contextual insensitivity remain relevant risks of these systems [12,13,14]. Direct comparative evidence against the physician-written standard, under real-world conditions and within the constraints of European data protection regulations (GDPR), is scarce.

Within the DCGIAG project—developed by the Fundació Privada Hospital Asil de Granollers (FPHAG) together with the technology company INNEX—a generative AI tool for hospital discharge reports (IAIA) was designed and evaluated, combining prior local anonymisation with cloud-based generation. The present study directly compares, within the same consecutive series of patients, the quality of reports generated by IAIA against those drafted by the responsible physician (INF), with the aim of assessing whether AI-generated documentation improves formal quality, clinical structure, and perceived utility for care continuity, without assuming clinical equivalence or substitution of medical judgement.

## 2. Materials and Methods

### 2.1. Study Design

A retrospective, paired expert-evaluation study was designed to assess whether AI-assisted discharge reports were comparable to—or better than—the physician-written standard under real-world conditions. Each clinical case was presented to the evaluators in two explicitly identified versions: the conventional physician-written discharge report (INF), considered the current standard of care comparator, and the AI-assisted discharge report (IAIA). This origin labelling was a retrospective evaluation-design choice, made when reports already generated during routine care (INF) or produced retrospectively by IAIA were subsequently assembled for expert rating; it was not a prospective intervention in the clinical process itself. Evaluators were informed of each report’s origin so that they could anchor their judgement to a known reference, consistent with the study’s retrospective, non-interventional design. We acknowledge, however, that masking the origin labels while preserving the content of each report would have been feasible and would have strengthened the internal validity of the comparison; this trade-off is discussed further as a limitation in Section 4.1. The INF reports were collected with the authorisation of the clinical departments under a retrospective design; individual clinician consent was not required given the anonymised and non-interventional nature of the study. The IAIA reports were generated retrospectively using exactly the same clinical source data—including structured fields such as allergies and medication—that was available at the precise moment of the original discharge, with no subsequent additions, corrections, or updates; IAIA therefore did not have access to more complete or later-consolidated information than the physician had when drafting the INF report. All clinical data were anonymised on a local server at the centre before any external processing, adopting a GDPR-oriented approach [15]; this reflects an architectural design principle and does not by itself constitute formal certification of regulatory compliance.

### 2.2. Setting and Discharge Report Writing Systems

The study was conducted at the Fundació Privada Hospital Asil de Granollers (Hospital General de Granollers, University Hospital). Two systems were compared:

- INF (control). Discharge report drafted entirely by the responsible physician without technological assistance, following the institutional template; considered the conventional comparator for this evaluation.
- IAIA (intervention). Discharge report generated by a generative AI tool with a hybrid architecture (local anonymisation on the hospital server and subsequent cloud-based processing by INNEX), described in detail in How the IAIA Works Section.

#### How the IAIA Works

IAIA is an application based on generative artificial intelligence designed to optimise hospital document management through the automated creation of proposed discharge reports. The system retrieves and integrates the patient’s clinical information, including the emergency department report, the clinical course notes, the ICU discharge report, and the surgical intervention report, as applicable.

Patient clinical information is anonymised on a local hospital server by a hybrid engine combining deterministic linguistic rules and clinical-domain-specific regular expressions, NLP techniques for contextual entity recognition, and the gpt-oss-120b language model (an open-weight model published by OpenAI), the latter supporting detection of complex entities and indirect references (e.g., relatives’ names or specific locations) not reliably identifiable through rules alone. No retrained or fine-tuned model is used; customisation resides in the rule engine itself. The engine detects and anonymises, among other elements, patient names and surnames, clinical record numbers, national identity documents (DNI) and personal identification codes (CIP), contact details (telephone, postal address, email), and indirect or cross-referenced identifiers. This step ensures no identifiable data leaves the centre’s perimeter. The anonymised information is then transmitted via a Microsoft Azure API to INNEX’s cloud infrastructure, where GPT-4.1 (OpenAI) generates a structured draft discharge report from a prompt with role instructions, an institutional output template, and writing constraints. The draft is returned to the hospital information system, where the responsible physician validates it—corroborating or modifying the text per clinical judgment before signing. This model reduces drafting time without compromising clinical responsibility.

### 2.3. Sample and Case Distribution

One hundred and twenty clinical cases from nine hospital departments were included, yielding a total of 240 reports. The distribution by department is shown in Table 1. No formal a priori sample size calculation was performed; the sample was determined by availability within the study period. As descriptive information only, a post hoc power analysis for the Wilcoxon signed-rank test, based on the observed effect size (r ≈ 0.76) and α = 0.05, indicated that the achieved power exceeded 0.99; this post hoc calculation is reported for transparency only and does not compensate for, or justify, the absence of an a priori sample size calculation (see Section 4.1).

### 2.4. Evaluation Instrument

The evaluation instrument was informed by the regulatory framework of Royal Decree 1093/2010 (Minimum Data Set for Clinical Reports in the National Health System, MDSCR) [16] and the international patient safety standards established by the Joint Commission International, as well as quality criteria for hospital discharge reports established in the literature [1,2,17]. No formal item-by-item mapping or independent audit of this alignment was performed: the correspondence with these regulatory and bibliographic sources is conceptual, not empirically validated for the present instrument. No prior study was identified that validated a discharge-report-specific quality rubric using a comparable multidimensional, weighted structure; the closest precedents are general chart-audit instruments for care-continuity information [1,2], rather than instruments purpose-built for AI-assisted documentation. A recent systematic review of LLM performance on clinical data extraction, although centred on pathology reports, illustrates the broader methodological standards increasingly expected in this area, including structured assessment of risk of bias and reporting completeness [18].

Each report was assessed across 13 structural clinical dimensions: reason for admission, family history, allergies and intolerances, patient baseline status, medical history, prior treatment, current illness, physical examination, diagnostic and complementary tests, clinical course, procedures, principal diagnosis, and other diagnoses (secondary diagnoses). Each dimension was scored on an ordinal scale from 1 to 3 (1 = poor, 2 = adequate, 3 = excellent), following a pre-established rubric with explicit descriptors per score level and a pre-assigned relative weight per dimension (see Supplementary Material S1). This 3-point scale mirrors the coarse-grained categories (deficient/adequate/excellent) commonly used in clinical documentation quality audits, prioritising inter-evaluator comprehensibility over finer discrimination; the ceiling effect observed for prior treatment and physical examination (Section 3.1) is a plausible consequence of this restricted range. Each report also received an overall rating on a scale from 1 to 10 and a free-text qualitative comments field.

Before the definitive study, the rubric underwent a pilot phase with reports not included in the final analysis, allowing the comprehensibility of the descriptors to be verified and points of uncertainty to be refined. This phase was qualitative in nature: the number of pilot cases, inter-rater agreement observed during piloting, and specific descriptor revisions were not systematically recorded and are therefore not reported quantitatively (see Section 4.1).

### 2.5. Evaluators

Ten primary care physicians in active clinical practice carried out the evaluation independently. Primary care physicians were selected because they are one of the main clinical recipients of the discharge report and must interpret its content to ensure care continuity following hospitalisation. Each evaluator was randomly assigned 12 patients with their two corresponding reports (INF and IAIA), totalling 24 reports assessed per professional. The IAIA-generated reports were delivered exactly as processed by the platform, without any subsequent intervention. To allow paired comparisons and ensure a homogeneous distribution of workload, assignment was made at the complete case level, always keeping both reports for the same patient assigned to the same evaluator. All ten evaluators practised at different primary care centres across the region and were external to both the hospital where the study was conducted and to INNEX, with no employment, contractual, or financial relationship with either institution. Each clinical case was rated by a single assigned evaluator; no case was independently double-rated by a second evaluator, so inter-rater reliability could not be estimated, and evaluator-level effects cannot be statistically separated from report-level effects in the present design (see Section 4.1).

### 2.6. Statistical Analysis

Given the ordinal nature of the variables and the impossibility of assuming normal distributions, the Wilcoxon signed-rank test was applied to all comparisons between systems. Pairs were constructed at the case level (each INF report paired with the corresponding IAIA report for the same patient), thereby controlling for inter-individual variability. The significance level was set at α = 0.05. Given the multiplicity of the 13 per-dimension comparisons, a Bonferroni correction was applied post hoc to control the family-wise error rate (adjusted significance threshold α = 0.05/13 ≈ 0.0038); nominal *p*-values are reported together with their significance status after this correction, and per-dimension findings that do not survive the correction are interpreted as trends rather than confirmed differences. The primary endpoint of the study was the overall rating (1–10), which was not subject to multiple testing. Artificial intelligence assistance (Claude, Anthropic) was used to support statistical calculation and verification. All results were independently reviewed and validated by the authors, who take full responsibility for the accuracy and interpretation of all statistical analyses reported in this manuscript. The primary inferential comparison is the overall rating. Alongside the p-value for the comparison of the overall rating, the effect size was calculated using the paired rank-biserial correlation (r), interpreted according to conventional thresholds (r < 0.1 trivial; 0.1–0.3 small; 0.3–0.5 moderate; ≥0.5 large). Continuous variables are described as mean ± standard deviation, and ordinal and 1–10 scale variables also as median and interquartile range (Q1–Q3). The overall rating categories were defined a priori as low (≤5), medium (6–7), and high (≥8), a study-specific convention based on the distribution of the 1–10 scale rather than an externally validated clinical threshold, since no prior study establishes standard cut-points for this instrument. The analysis was performed with Python SciPy 1.11.

Each of the 240 evaluated reports could receive at most one free-text qualitative comment; when a single comment addressed more than one distinct aspect of report quality, each aspect was coded as a separate observation, which explains why the total of 247 coded observations exceeds the number of reports. These free-text observations were analysed through an inductive reflexive thematic analysis, following a contextualist epistemological position—a form of subtle realism intermediate between naïve realism and radical constructionism—under which evaluators’ comments are treated as reflecting meaningful, if perspective-dependent, features of report quality rather than either objective facts or purely constructed narratives. Two reviewers, neither of whom had any role in the design or development of the AI system under evaluation, independently read all comments, generated open codes from the material itself, and iteratively grouped the codes into categories and themes; coding was considered to have reached saturation when a complete pass through the remaining uncoded material produced no new themes. After a first round of independent coding, discrepancies in code or theme assignment were discussed jointly and resolved by consensus; no formal inter-rater agreement statistic was computed for this qualitative coding step. Exact per-theme frequency counts were not systematically tabulated during the original coding process; therefore reports themes qualitatively were ordered by the coders’ impression of relative prevalence (the most frequently mentioned weakness is explicitly flagged as such), rather than as quantified counts, and anonymised verbatim quotations are preserved as illustrative evidence.

### 2.7. Ethics and Data Protection

All clinical cases were anonymised on the centre’s local infrastructure before being processed by any system, both for the study evaluation and for the generation of AI-assisted reports. This prior anonymisation ensures that no identifiable patient data is transmitted to external infrastructures, substantially reducing the risk of disclosure of identifiable clinical information, in alignment with the General Data Protection Regulation (GDPR) [15]. The physicians whose discharge reports (INF) were included as the comparator were not individually informed of the study; their inclusion was authorised at departmental level under the retrospective, non-interventional, and fully anonymised design certified by the Clinical Research Ethics Committee (CEIm) of the Fundació Privada Hospital Asil de Granollers, and no clinician-identifiable information was processed or disclosed at any stage.

## 3. Results

### 3.1. Analysis by Clinical Dimensions

**Figure 1:**
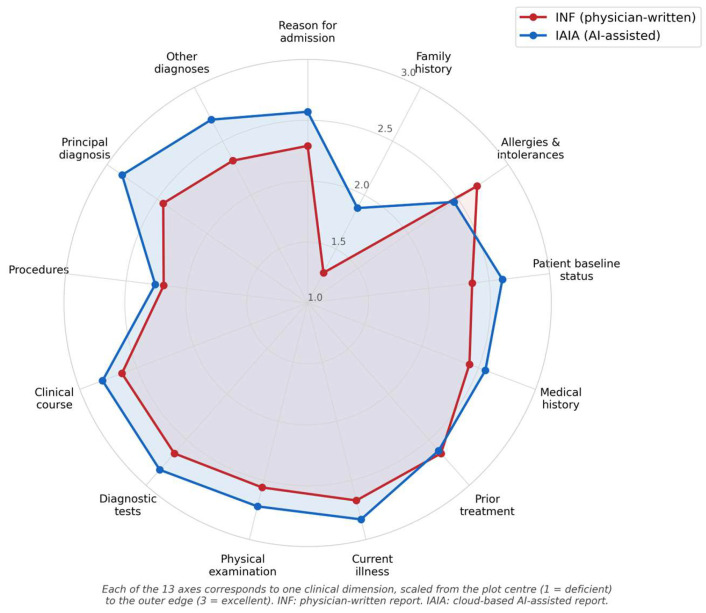
Mean score per clinical dimension (scale 1–3) for each system, represented as a radar chart.

Mean scores by dimension and Wilcoxon test p-values for the paired comparison are shown in Figure 1 and Table 2. IAIA was nominally superior to INF in 9 of the 13 dimensions; INF was superior in one dimension (allergies and intolerances); and three dimensions showed no significant differences between systems (prior treatment, physical examination, procedures). Because 13 independent per-dimension comparisons were performed, a Bonferroni correction was applied (adjusted α = 0.05/13 ≈ 0.0038); after correction, six of the nine nominally IAIA-favourable dimensions and the allergies and intolerances comparison remained statistically significant, while medical history, current illness, and diagnostic tests no longer reached the adjusted threshold and are reported as trends rather than confirmed differences (see Table 2).

The most relevant gains for IAIA were observed in family history (1.88 vs. 1.28; *p* < 0.001; with 39.2% of reports rated excellent versus 13.2% for INF) and principal diagnosis (2.85 vs. 2.44; *p* < 0.001), reflecting better diagnostic hierarchy; improvements were also significant, after Bonferroni correction, in reason for admission, baseline status, clinical course, and other diagnoses. Medical history (*p* = 0.040), current illness (*p* = 0.005), and diagnostic tests (*p* = 0.006) were nominally significant but did not survive correction for multiple comparisons and are reported as trends rather than confirmed differences. INF retained an advantage only in allergies and intolerances (2.69 vs. 2.46; *p* = 0.002), an area in which IAIA tended to use standardised formulas that did not explicitly state whether the absence of allergies had been actively verified. The non-significant dimensions show distinct patterns: prior treatment (2.65/2.62) and physical examination (2.56/2.72) reached scores close to the maximum of the scale, suggesting a ceiling effect; by contrast, procedures (2.19/2.26) were further from the maximum, so the absence of statistical significance in this dimension most likely reflects greater variability rather than scale saturation. Given that allergies and intolerances constitute critical information for pharmacological safety, this result should be interpreted as a specific alert regarding the need for active clinical verification in any AI-assisted workflow.

### 3.2. Overall Rating

Descriptive statistics for the overall rating (scale 1–10) are summarised in Table 3. IAIA achieved the highest mean (8.14/10) versus 7.30/10 for INF. IAIA concentrated 75.0% of its reports in the high-quality range (≥8) compared with 51.2% for INF, while the proportion of reports in the low range (≤5) fell from 9.9% with INF to 3.3% with IAIA, a relative reduction of 67%.

**Figure 2:**
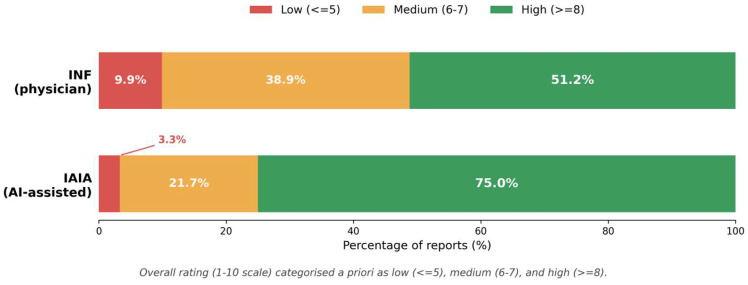
Percentage distribution of the overall rating (1–10) by pre-established ranges for each system (low: ≤ 5; medium: 6–7; high: ≥ 8).

Categorisation of the overall rating into pre-specified ranges (Figure 2) confirmed that IAIA concentrated three-quarters of its reports in the high range, INF presented a more balanced profile with approximately half of its reports in the high range, and the low range was reduced from one in every ten reports with INF to one in every thirty with IAIA.

The Wilcoxon signed-rank test confirmed that IAIA was statistically superior to INF in the overall rating, with a large effect size (Table 4). When summing the 13 ordinal dimensions (maximum 39 points), IAIA obtained 33.7 versus 31.1 for INF, with a difference that was also highly significant (*p* < 0.0001) and of a similarly large magnitude (r ≈ 0.74, 95% CI 0.65–0.81; Table 4). The convergence between the global result and the sum of dimensions indicates that IAIA’s advantage is reflected both in the holistic perception of the report and in its structural completeness.

As a supplementary consistency check, the advantage of IAIA over INF in the overall rating was observed in 7 of the 10 evaluators (mean difference range: +0.42 to +2.92); the three evaluators who rated INF higher showed smaller differences (range: −0.24 to −0.74), suggesting that the overall result is not driven by a small subset of evaluators. This check addresses the direction of the effect across evaluators and should not be read as a substitute for a formal inter-rater reliability estimate: since each case was scored by only one evaluator, it is not possible to assess whether two evaluators would assign the same absolute score to the same report (see Section 4.1).

### 3.3. Qualitative Analysis

Two hundred and forty-seven free-text qualitative observations provided by the 10 evaluators were analysed through reflexive thematic analysis. The recurring strengths and weaknesses identified for each system are summarised in Table 5.

## 4. Discussion

In this paired, non-blinded expert-evaluation study conducted under real-world conditions, AI-assisted discharge reports not only met but exceeded the documentary quality of physician-written reports (8.14 vs. 7.30 out of 10; *p* < 0.0001; r ≈ 0.76, large effect size), nominally outperforming current clinical documentation practice in nine of the 13 structured dimensions, only six of which remained significant after Bonferroni correction for multiple comparisons, and concentrating three-quarters of their reports in the high-quality range. These findings exceed the initial comparative expectations of the study; however, given the non-blinded, single-centre design, they should be interpreted as evidence of higher expert-rated documentary quality under the evaluation conditions employed, rather than as a demonstration of overall superiority of AI-assisted documentation over the current standard. The magnitude and direction of these findings are consistent with results published by Van Veen and colleagues for LLMs adapted to clinical text summarisation tasks [5] and with the documentary quality improvements observed in early deployments of ambient AI scribes [9], placing our results within the expected range for this technology. As noted in the Introduction, however, the Van Veen et al. benchmark was task-specific and discharge-summary-focused studies have reported more mixed results [6,7,8]; our finding of a documentary quality advantage should therefore be interpreted as consistent with, rather than a direct replication of, that broader body of evidence.

These results should not be interpreted as a demonstration of clinical superiority of AI over the physician, but rather as evidence that a structured generative system can produce more complete, homogeneous, and easily interpretable discharge drafts. The principal contribution of the model does not lie in replacing clinical judgement, but in reducing documentary variability and supporting the professional in synthesis and drafting tasks. The 0.84-point difference in the overall rating (8.14 vs. 7.30 on a 1–10 scale) should be understood strictly as a gain in documentary quality under the evaluation framework used here; on its own, it does not demonstrate an improvement in real clinical outcomes, since no readmission, medication-error, or other patient-relevant endpoints were measured in this study.

Three findings are clinically relevant. First, IAIA’s gains were concentrated in dimensions where conventional physician writing has shown the greatest weakness—family history, diagnostic hierarchy, and structured listing of secondary diagnoses—precisely those areas where the narrative effort competes with clinical workload pressure; the qualitative analysis (Section 3.3) similarly identified discharge recommendations as a recurring weakness of physician-written reports. Second, INF retained a small but significant advantage in allergies and intolerances, an area critical for safety that depends on the physician’s active reconciliation with the patient and does not admit plausible but unverified formulations. This is consistent with the warning by Goodman and colleagues that AI-generated clinical summaries require far more than accuracy and demand robust verification of safety-critical content [12]. Third, the absence of significant differences in prior treatment and physical examination suggests that IAIA’s advantage operates primarily over dimensions requiring synthesis and structuring of dispersed information, while these dimensions, fed by structured EHR data or by direct physician-patient interaction, show comparable performance; in procedures, the non-significance most likely reflects intrinsic variability of the dimension rather than scale saturation.

The qualitative analysis provides a complementary perspective. Recurring criticisms of INF—unexplained acronyms, spelling and typographical errors, language mixing, and absent or generic discharge recommendations—are problems that simple support measures (writing guidelines, structured templates, automated spell-checking) could partially address. The recurring weakness of IAIA—standardised allergy formulas that do not explicitly state whether active verification was performed—represents a specific clinical safety risk that cannot be addressed by prompt design alone: allergy and intolerance data must not be inferred by the model but must be sourced from a verified structured field or explicitly flagged as pending mandatory physician validation. Both observations reinforce a hybrid implementation model in which AI drafts the proposal and the clinician validates and refines critical content, rather than a complete replacement [13,19].

A distinctive contribution of this study is demonstrating that generative AI can be deployed in a European hospital setting under a GDPR-oriented architecture based on prior local anonymisation without compromising documentary quality, offering a template that other public hospitals operating under similar data-protection constraints could adapt.

### 4.1. Limitations

This study has several limitations. It is a single-centre study, and small per-department samples preclude inference at the speciality level. No a priori sample size calculation was performed; cases were included based on availability rather than a pre-specified target. The post hoc power analysis reported in Section 2.3 describes the sample actually obtained and must not be read as a retrospective justification for the absence of an a priori calculation.

Both reports were explicitly identified to evaluators, which is a major limitation. This design allowed IAIA to be judged against real-world standard-of-care documentation, but since the rubric scores each report independently, masking origin (revealed only at analysis) would have been feasible and would have permitted a fully blinded comparison; this design choice should not be treated as methodologically equivalent to open-label trials, where blinding is precluded by practical constraints rather than by choice. Unmasked origin raises the risk of automation bias—evaluators rating AI-generated content more favourably once its origin is known, a well-documented, unidirectional effect in IAIA’s favour; a counteracting familiarity bias favouring physician-written reports is plausible but more speculative. This bias may have contributed to the large observed effect size (r ≈ 0.76); a blinded, randomised-order replication is a priority for future research.

Each case was scored by a single evaluator, which is also a major limitation: because no case was scored by more than one evaluator, evaluator-level effects (idiosyncratic scoring tendencies, differing familiarity with the rubric) cannot be statistically separated from true report-level quality differences. The evaluator-consistency check (Section 3.2) confirms directional consistency but not absolute agreement and cannot substitute for a formal reliability coefficient (e.g., ICC or weighted kappa); double-rating a subset of cases would strengthen future designs. The rubric itself has not undergone formal psychometric validation—no content validity index, internal consistency statistic, or construct validity testing was performed; the instrument rests on face validity and a single qualitative pilot round rather than on documented psychometric properties. Its alignment with Royal Decree 1093/2010 and Joint Commission International criteria has likewise not been independently audited. These gaps limit how confidently absolute score differences can be attributed to true documentary quality rather than instrument-level noise.

Deferred clinical outcomes (30-day readmission, medication errors, primary care satisfaction) were not assessed, so documentary quality gains should not be assumed to translate into clinical or operational benefit. Review order was not randomised, so an order effect cannot be excluded, and the thematic analysis of free-text comments (Section 2.6) reports relative prevalence qualitatively rather than by exact frequency count.

Drafting time was not systematically assessed here; the commonly cited 30–45-minute saving per report comes from a separate prior proof-of-concept (Cardiology and Pulmonology, FPHAG and INNEX) [20] and should not be conflated with the documentary quality results reported above.

### 4.2. Clinical Implications and Future Directions

The findings of this study open a pathway for future research that must go beyond formal documentary quality: prospective, multicentre studies are needed to evaluate the impact of IAIA on deferred clinical outcomes, such as reduction in readmissions, medication errors, and primary care satisfaction, as well as on the time actually saved by professionals in drafting clinical reports. Regarding implementation implications, the data support a supervised hybrid model (human-in-the-loop) in which AI generates the initial draft to reduce the administrative burden, while the clinician refines the narrative and mandatorily validates safety-critical content, particularly allergy and intolerance information, which requires verification against structured fields rather than model-based inference. Hospital integration requires replicating the architecture of this project, maintaining prior local anonymisation of data as a non-negotiable requirement for a GDPR-oriented deployment in the cloud.

## 5. Conclusions

In this retrospective, single-centre non-blinded paired evaluation, discharge reports generated by a cloud-based generative AI tool (IAIA) received higher expert-rated documentary quality scores than those drafted by the physician in most of the dimensions examined, with the widest differences in sections requiring information synthesis and structuring. The physician-written report retained an advantage only in one safety-critical dimension (allergies and intolerances): allergy and intolerance information must not be inferred by the model but sourced from verified structured fields or explicitly flagged as pending mandatory physician validation. The architecture employed—prior local anonymisation with cloud-based generation—integrates AI into the clinical workflow using a GDPR-oriented approach, although full regulatory compliance must be specifically audited before any deployment. Generalisation of the documentary quality findings to other centres, models, or time periods requires multicentre prospective studies incorporating deferred clinical outcomes—such as readmissions, medication errors, and follow-up adherence—before any claim of clinical benefit, as opposed to documentary quality benefit, can be made.

## Figures and Tables

**Table 1:** Distribution of cases by clinical department.

Department	INF (*n*)	IAIA (*n*)	Total
Gastroenterology	5	5	10
Cardiology	6	6	12
Acute Geriatrics	35	35	70
Internal Medicine	43	43	86
Neurology	6	6	12
Orthogeriatrics	6	6	12
Pulmonology	6	6	12
Subacute Care	7	7	14
Intensive Care Unit	6	6	12
TOTAL	120	120	240

**Table 2:** Mean score per clinical dimension (scale 1–3) and Wilcoxon *p*-values.

Dimension	INF	IAIA	Δ	*p*-Value	Direction
Reason for admission	2.29	2.57	+0.28	<0.001	IAIA superior
Family history	1.28	1.88	+0.60	<0.001	IAIA superior
Allergies and intolerances	2.69	2.46	−0.23	0.002	INF superior
Patient baseline status	2.36	2.61	+0.25	<0.001	IAIA superior
Medical history	2.42	2.56	+0.14	0.040	IAIA superior (ns, Bonferroni)
Prior treatment	2.65	2.62	−0.03	ns	No differences
Current illness	2.67	2.83	+0.16	0.005	IAIA superior (ns, Bonferroni)
Physical examination	2.56	2.72	+0.16	0.052 (ns)	No differences
Diagnostic tests	2.65	2.83	+0.18	0.006	IAIA superior (ns, Bonferroni)
Clinical course	2.63	2.80	+0.17	0.001	IAIA superior
Procedures	2.19	2.26	+0.07	ns	No differences
Principal diagnosis	2.44	2.85	+0.41	<0.001	IAIA superior
Other diagnoses	2.32	2.70	+0.38	<0.001	IAIA superior

ns = not significant. Wilcoxon signed-rank test for paired samples. Given the 13 per-dimension comparisons performed, a Bonferroni correction was applied (adjusted significance threshold α = 0.05/13 ≈ 0.0038).

**Table 3:** Descriptive statistics of the overall rating (scale 1–10).

Statistic	INF (Physician)	IAIA (AI-Assisted)
Number of reports	120	120
Mean	7.30	8.14
Median	8.0	8.0
IQR (Q1–Q3)	6.0–8.0	7.75–9.0
Standard deviation	1.33	1.20
Minimum	4.0	3.0
Maximum	10.0	10.0
% ≥8 (high)	51.2%	75.0%
% ≤5 (low)	9.9%	3.3%

**Table 4:** Wilcoxon signed-rank test and effect size: INF vs. IAIA.

Comparison	Paired n	W	*p*-Value	r (Paired rb)	Direction
Overall rating (1–10)	120	867.0	<0.0001	≈0.76 (large) [95% CI 0.67–0.83]	IAIA superior
Sum of 13 dimensions (max 39)	120	939.5	<0.0001	≈0.74 (large) [95% CI 0.65–0.81]	IAIA superior

r = paired rank-biserial correlation; values derived from the W statistic. 95% confidence intervals were obtained via the Fisher z-transformation of r.

**Table 5:** Recurring strengths and weaknesses identified in the thematic analysis of the 247 qualitative observations.

	INF	IAIA
Strengths	• Coherent clinical reasoning, especially in current illness and clinical course • Specific and verified allergy recording • Human and contextual sensitivity in complex cases (end of life, advanced chronic illness)	• Structured and natural writing with coherent narrative articulation • Informational completeness across all template sections • Systematic inclusion of therapeutic plan, follow-up recommendations, and re-consultation criteria • Correct hierarchy of principal and secondary diagnoses
Weaknesses	• Unexplained acronyms (the most frequent criticism) • Spelling and typographical errors • Language mixing (Catalan/Spanish) within the same report • Incomplete listing of procedures and secondary diagnoses • Absent or generic discharge recommendations	• Use of standardised allergy formulas that do not explicitly state whether their absence has been actively verified

## Data Availability

The anonymised evaluation dataset used in this study is not publicly available due to institutional data governance constraints. It may be made available upon reasonable request to the corresponding author, subject to applicable regulations.

## Supplementary Materials

The following supporting information can be downloaded at: https://www.mdpi.com/article/10.3390/healthcare14152290/s1, Supplementary Material S1: Complete Evaluation Rubric for Hospital Discharge Report Quality Assessment.
