# Supplementary material for: Expert-Rated Documentary Quality of AI-Assisted Hospital Discharge Reports: A Retrospective Paired Comparison with Physician-Written Reports
Source: Healthcare (Basel). 2026 Jul 29;14(15):2290. doi: 10.3390/healthcare14152290 (PMC13464595; doi:10.3390/healthcare14152290)
Supplement: Supplementary file 1 [file healthcare-14-02290-s001.zip › healthcare-4408995-supplementary.pdf]

Supplementary Material S1. Complete Evaluation Rubric for Hospital Discharge Report Quality Assessment.

Regulatory framework: Royal Decree 1093/2010 (Minimum Data Set for Clinical Reports in the National Health System, Spain) and Joint Commission International patient safety standards. The rubric underwent a pilot phase with reports not included in the final analysis to verify descriptor comprehensibility.

Article: Expert-rated documentary quality of AI-assisted hospital discharge reports: a retrospective paired comparison with physician-written reports

DCGIAG Project · Fundació Privada Hospital Asil de Granollers (FPHAG)

Clinical Dimension	Weight	Scoring Descriptors (1 = Poor · 2 = Adequate · 3 = Excellent)
**Reason for admission**	4%	Excellent (3): reason clearly stated, clinically justified and consistent with the reported course. Adequate (2): reason present but lacking detail. Poor (1): absent, ambiguous or inconsistent with the clinical record.
**Family history**	4%	Excellent (3): clinically relevant family history well described and linked to the current episode. Adequate (2): some family history recorded. Poor (1): incomplete or absent.
**Allergies and intolerances**	2%	Excellent (3): complete and verified list, or explicit statement confirming active verification of the absence of allergies. Adequate (2): incomplete list or generic formula without verified status. Poor (1): not recorded.
**Patient baseline status**	6%	Excellent (3): functional and cognitive status described with precision (e.g., Barthel index, cognitive level). Adequate (2): basic description present. Poor (1): vague or absent.
**Medical history**	12%	Excellent (3): comprehensive list with chronology and clinical context. Adequate (2): incomplete. Poor (1): superficial or absent.
**Prior treatment**	6%	Excellent (3): complete medication list with doses and frequency. Adequate (2): partial list without doses or with omissions. Poor (1): incomplete or absent.
**Current illness**	10%	Excellent (3): chronological, coherent description of the presenting complaint and its evolution prior to admission. Adequate (2): partial description. Poor (1): unclear, absent or incoherent.
**Physical examination**	6%	Excellent (3): complete, structured by systems or body regions. Adequate (2): schematic but present. Poor (1): incomplete or clinically insufficient.
**Diagnostic and complementary tests**	10%	Excellent (3): all relevant tests included with adequate interpretive information. Adequate (2): main tests mentioned with some data or interpretation missing. Poor (1): relevant information absent or not reported.

**Clinical course (primary criterion)**	20%	Excellent (3): chronological, coherent and reasoned description of the in-hospital course; reflects clinical follow-up, treatment response, decision-making and medical judgement. Adequate (2): evolution described but with little detail or analytical reasoning. Poor (1): superficial, disorganised or absent.
**Procedures**	6%	Excellent (3): complete list of procedures performed, or explicit statement that no procedures were performed. Adequate (2): partial mention. Poor (1): not recorded.
**Principal diagnosis**	8%	Excellent (3): diagnosis precise, hierarchically primary, and consistent with the clinical presentation. Adequate (2): coherent but poorly substantiated or not clearly primary. Poor (1): unclear, absent or erroneous.
**Other diagnoses**	6%	Excellent (3): complete and hierarchically organised list of secondary diagnoses. Adequate (2): some secondary diagnoses listed. Poor (1): absent or grossly incomplete.

Each of the 13 clinical dimensions was scored on an ordinal scale from 1 to 3 (1 = Poor, 2 = Adequate, 3 = Excellent) according to the descriptors below. The weight column indicates the relative clinical relevance assigned to each dimension in the design of the rubric; weights informed the scoring descriptors but were not incorporated into the statistical analysis, which was based on the unweighted sum of the 13 ordinal scores (maximum 39 points).
